# Anastrozole (Arimidex ™) – an aromatase inhibitor for the adjuvant setting?

**DOI:** 10.1054/bjoc.2001.1983

**Published:** 2001-02-01

**Authors:** A U Buzdar

**Affiliations:** Department of Breast Medical Oncology, MD Anderson Cancer Center, University of Texas, 1515 Holcombe Bonlevard, Box 56, Houston, Texas 77030, USA

**Keywords:** anastrozole, aromatase inhibitor, tamoxifen, advanced breast cancer, adjuvant therapy, postmenopausal

## Abstract

Anastrozole (Arimidex™) is a third-generation aromatase inhibitor which has been shown to possess superior efficacy and tolerability over established endocrine agents in advanced breast cancer. Inhibition of aromatase prevents the conversion of androgen substrates to oestrogen, its sole source in postmenopausal women, thereby leading to regression of hormone-sensitive breast carcinomas. Clinical pharmacology data indicate that anastrozole is a potent aromatase inhibitor, providing near-maximal suppression of serum and intratumoural oestrogens to below detectable levels. Anastrozole may offer greater selectivity compared with other aromatase inhibitors, being without any intrinsic endocrine effects and with no apparent effect on the synthesis of adrenal steroids. It is well tolerated and has a convenient once-daily dosing regimen, ensuring maximum patient compliance. A major clinical programme has demonstrated that anastrozole is superior to the standard endocrine therapy, tamoxifen, for the first-line treatment of postmenopausal women with hormone-sensitive advanced breast cancer. Its superior efficacy in advanced disease, together with its improved tolerability and convenient dosage, make it a suitable agent to be assessed for the treatment of early breast cancer in postmenopausal women. This was investigated in the largest single adjuvant breast cancer study ever to be carried out, the ATAC (Arimidex, tamoxifen, alone or in combination) trial, which has now completed recruitment, with the first efficacy and safety data awaited. © 2001 Cancer Research Campaign


*British Journal of Cancer* (2001) **85**(Suppl 2), 6–10
© 2001 Cancer Research Campaign
doi: 10.1054/ bjoc.2001.1983, available online at http://www.idealibrary.com on IDEAL

### Anastrozole (Arimidex™) – an aromatase inhibitor for the adjuvant setting?

**AU Buzdar**

Department of Breast Medical Oncology, MD Anderson Cancer Center, University of Texas, 1515 Holcombe Bonlevard, Box 56, Houston, Texas 77030, USA

**Summary** Anastrozole (Arimidex™) is a third-generation aromatase inhibitor which has been shown to possess superior efficacy and tolerability over established endocrine agents in advanced breast cancer. Inhibition of aromatase prevents the conversion of androgen substrates to oestrogen, its sole source in postmenopausal women, thereby leading to regression of hormone-sensitive breast carcinomas. Clinical pharmacology data indicate that anastrozole is a potent aromatase inhibitor, providing near-maximal suppression of serum and intratumoural oestrogens to below detectable levels. Anastrozole may offer greater selectivity compared with other aromatase inhibitors, being without any intrinsic endocrine effects and with no apparent effect on the synthesis of adrenal steroids. It is well tolerated and has a convenient once-daily dosing regimen, ensuring maximum patient compliance. A major clinical programme has demonstrated that anastrozole is superior to the standard endocrine therapy, tamoxifen, for the first-line treatment of postmenopausal women with hormone-sensitive advanced breast cancer. Its superior efficacy in advanced disease, together with its improved tolerability and convenient dosage, make it a suitable agent to be assessed for the treatment of early breast cancer in postmenopausal women. This was investigated in the largest single adjuvant breast cancer study ever to be carried out, the ATAC (Arimidex, tamoxifen, alone or in combination) trial, which has now completed recruitment, with the first efficacy and safety data awaited. © 2001 Cancer Research Campaign

**Keywords**: anastrozole; aromatase inhibitor; tamoxifen; advanced breast cancer; adjuvant therapy; postmenopausal

### INTRODUCTION

The development of new aromatase inhibitors such as anastrozole represents a significant step forward in the clinical management of postmenopausal women with hormone-sensitive breast cancers. The evidence in support of anastrozole – a non-steroidal, orally administered aromatase inhibitor – as an effective antitumour agent is outlined here.

Anastrozole was one of the first agents to move immediately from phase I studies of clinical pharmacology into clinically evaluable phase III trials, due primarily to its proven effectiveness in reducing plasma oestrogen, a recognized surrogate marker for anti-aromatase activity. Large-scale trials have now shown that anastrozole shows significant benefits over established endocrine agents in both first- and second-line management of advanced breast cancer. Consequently, anastrozole is an effective alternative to tamoxifen for first-line treatment of advanced disease in postmenopausal women and is now available for such use in a number of different countries. Given this superior efficacy profile in the management of advanced disease, anastrozole may also provide advantages in early disease therapy, where long-term treatment may be required to prevent disease recurrence.

Since its introduction in 1973, tamoxifen has remained the endocrine therapy of choice for early breast cancer in postmenopausal patients (Early Breast Cancer Trialists' Collaborative Group, 1992, 1998) and has provided clinicians with an effective and well tolerated treatment. However, tamoxifen has a number of limitations, based primarily on its partial oestrogen agonist activity, which contribute directly to endometrial cell proliferation and rarely endometrial cancer (Fisher et al, 1998) and breast tumour stimulation. In contrast, aromatase inhibitors act by suppressing conversion of androgen substrates into oestrogen (the primary source of oestrogen in postmenopausal women), thereby reducing the incidence of the more serious side-effects associated with tamoxifen while maintaining efficacy for the management of hormone-sensitive breast cancer.

Primary prerequisites for effective alternative agents in the adjuvant or preventative settings include equivalent or superior efficacy and/or improved tolerability compared with tamoxifen, together with easy and convenient administration. Tolerability assumes a much greater importance in the adjuvant setting, where prolonged therapy requires an agent with efficacy but minimal side-effects.

### EARLY AROMATASE INHIBITORS

The first-generation aromatase inhibitor, aminoglutethimide, has been available for therapeutic use since the late 1970s. While clinical studies showed that aminoglutethimide is as effective as tamoxifen in advanced disease, its use was hampered by significant problems relating to its toxicity, lack of selectivity and inconvenient dosing regimen (Smith et al, 1982; Gale et al, 1994). Subsequently, formestane – a second-generation steroidal aromatase inhibitor – became available in the early 1990s but was not an ideal replacement, given its requirement for intramuscular administration every 2 weeks, and associated injection-site reactions (Johnston and Metcalf, 1984). Both agents were also less potent in terms of aromatase inhibition and oestrogen suppression than the new-generation compounds.

### THIRD-GENERATION AROMATASE INHIBITORS

The limitations of aminoglutethimide and formestane provided the incentive to develop new-generation aromatase inhibitors. These include the non-steroidal agents anastrozole and letrozole, and the oral steroidal aromatase inhibitor exemestane. All are currently available for therapeutic use and are indicated as second-line treatments for postmenopausal women with advanced breast cancer who have progressed following first-line treatment with anti-oestrogens (tamoxifen). Anastrozole and letrozole are now also available for first-line treatment in these patients. While each one provides greater selectivity and more potent inhibition of aromatase compared with aminoglutethimide or formestane, clinically relevant differences have been reported between them in terms of their pharmacokinetic and pharmacodynamic profiles. These differences will ultimately influence choice not only in the treatment of advanced disease but also for their potential use in the adjuvant setting.

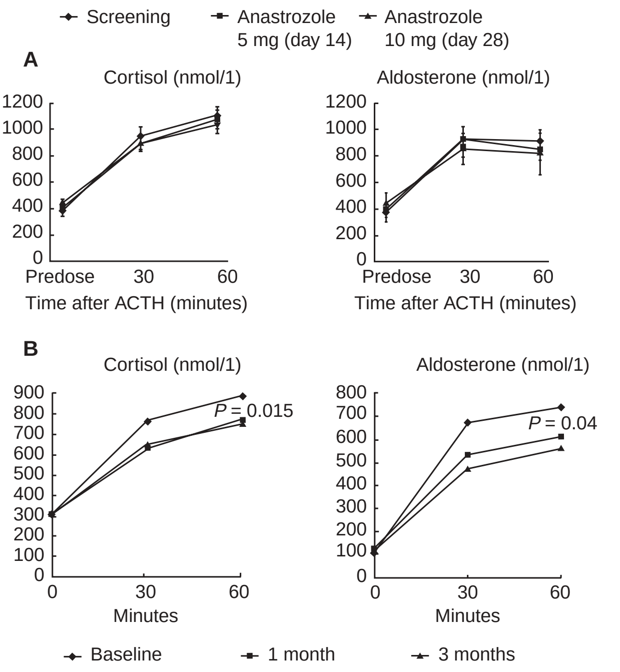

**Figure 1** Impact of (**A**) anastrozole (5 and 10 mg/day) and (**B**) letrozole (2.5 mg/day) on adrenal steroidogenesis: ACTH-stimulated results ((**A**) reproduced with kind permission of Kluwer Academic Publishers Boston from Plourde et al, 1994; (**B**) reproduced with kind permission of Elsevier Science Ltd from Bajetta et al, 1999)

While the three aromatase inhibitors are generally considered to be similar, differences exist in their clinical pharmacology. These differences are shown in Table 1. The profile for anastrozole is superior to the others in terms of:

- Effective dose
- Time to achieve steady-state levels and maximal oestrogen suppression (Plourde et al, 1995)
- Elimination half-life (Yates et al, 1996)
- Inhibition of intratumoural aromatase activity (Geisler et al, 1999).

In terms of overall selectivity and based on the available published data, anastrozole may be considered to be the most selective of the third-generation aromatase inhibitors in the clinical setting. There is no blunting of the response to adrenocorticotropin hormone (ACTH) stimulation, even when administered at 10 times the normal clinical dose of anastrozole over a period of 4 weeks, suggesting that anastrozole therapy does not interfere with adrenal steroidogenesis (Plourde et al, 1995) (Figure 1a). In contrast letrozole, administered at the usual clinical dose (2.5 mg/day), has been shown to impact significantly on both basal and ACTH-stimulated cortisol and aldosterone levels (Bajetta et al, 1999) (Figure 1b). This observation may have significant clinical impact, particularly if patients are under acute stress and when the drug is administered for long periods of time. It is possible that adrenal suppression may limit letrozole's use in the adjuvant and preventative settings where long-term administration is necessary. Exemestane is a steroidal agent and as such possesses intrinsic hormonal activities, thus behaving similarly to a weak androgen (Bajetta et al, 1997; Jones et al, 1999; Michaud and Buzdar, 1999). The potential for unwanted androgenic side-effects, including weight-gain (Kauffman et al, 2000), may limit its long-term utility in the adjuvant and preventative settings.

### ANASTROZOLE IN ADVANCED BREAST CANCER

### Anastrozole as a second-line agent

Anastrozole was the first aromatase inhibitor to demonstrate significant survival benefits compared with the standard second-line agent, megestrol acetate, in the treatment of postmenopausal women with advanced breast cancer progressing after prior

**Table 1** Indirect comparison of dosage, pharmacokinetics and endocrine selectivity of the different aromatase inhibitors, anastrozole, letrozole and exemestane

| | Aromatase inhibitor | | |
|---|---|---|---|
| | Anastrozole | Letrozole | Exemestane |
| Daily clinical dose (oral, mg/day) | 1 | 2.5 | 25 |
| Time to steady-state plasma levels (days) | 7 | 14–42 | 4 |
| Half-life | 40–50 hours | 2–4 days | 24 hours |
| Time to maximal oestradiol suppression (days) | 3–4 | 2–3 | by day 7* |
| Intratumoural activity | Yes | Yes | Yes |
| Androgen-like structure and properties | No | No | Yes |
| Effect on sex hormone binding globulin levels | None or decrease† ($P = 0.003$) | Increase ($P = 0.0001$) | Decrease |
| Effect on basal cortisol | None | None or decrease ($P < 0.003$) | None |
| Effect on basal aldosterone | None | None or increase ($P = 0.025$) | None |
| Effect on ACTH-stimulated cortisol | None | Decrease ($P = 0.015$) | ND |
| Effect on ACTH-stimulated aldosterone | None | Decrease ($P = 0.04$) | ND |
| Ratio of therapeutic dose that affects cortisol or aldosterone (x clinical dose) | > 10 | 1 | > 32 |

ND = no data; *samples taken at 7-day intervals; †observed in male subjects only

*© 2001 Cancer Research Campaign*

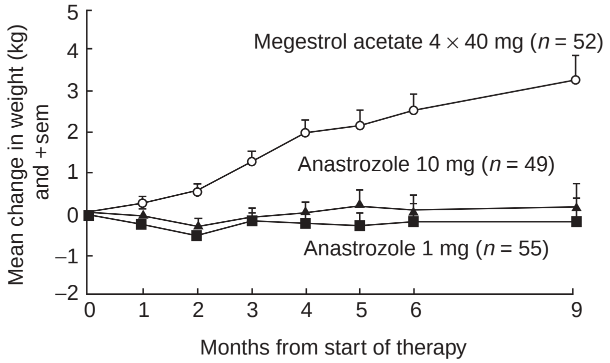

**Figure 2** Weight-gain over time. 9-month data update of anastrozole vs megestrol acetate (reproduced with kind permission of John Wiley and Sons Inc. from Buzdar et al, 1998)

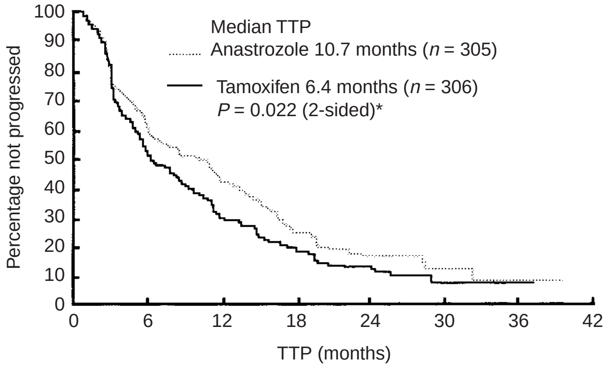

**Figure 3** Combined analysis of Kaplan–Meier curve of probability of TTP. Combined analysis of patients from trials 0027 (TARGET, European trial) and 0030 (North American trial). Analysis of patients known to be oestrogen-receptor-positive only (*based on retrospective analysis (Buzdar et al, 2000))

tamoxifen treatment. This was shown in two phase III clinical trials of identical design which compared the effects of two different daily doses of anastrozole (1 mg and 10 mg) with megestrol acetate (40 mg, four times daily) on time to progression and overall survival. In both studies, disease had progressed after prior tamoxifen therapy. Following initial assessment of efficacy at 6 months, anastrozole, 1 mg/day, was shown to be as effective as megestrol acetate, but was more tolerable with regard to weight-gain. The identical design of both trials allowed a prospectively planned combined analysis to be performed, thus strengthening the statistical power of the observations made in both trials, while also providing a survival update based on mature data at a median follow-up of 31 months, when over 60% of the patients had died. The combined analysis demonstrated that anastrozole significantly increased median survival compared with megestrol acetate (22.5 months in the megestrol acetate group vs 26.7 months in the anastrozole 1 mg/day group, $P < 0.025$) (Buzdar et al, 1998). No additional benefit was observed with anastrozole 10 mg (median survival 25.5 months, not significant), endorsing the choice of 1 mg as the clinical dose. Anticipated adverse events were pre-identified on the basis of the recognized pharmacology of both agents and were monitored in both treatment arms at 6 months. Of these anticipated events, no statistically significant differences were observed between either treatment arm, with the exception of weight-gain. This is a major problem associated with megestrol acetate therapy, with a significant proportion of patients complaining of an increase in weight ($P < 0.0001$) (Buzdar et al, 1998). Moreover, the weight-gain experienced by patients receiving megestrol acetate continued to increase over time. In contrast, no significant increase in weight was reported in patients receiving either dose of anastrozole (Figure 2). Anastrozole was subsequently approved by the US Food and Drug Administration for second-line use in the treatment of postmenopausal women with advanced breast cancer.

### Anastrozole as a first-line agent in advanced disease

A clinical programme has recently compared the efficacy and tolerability of anastrozole and tamoxifen in women with advanced breast cancer. This programme consisted of two large, multicentre, double-blind, double-dummy randomized trials (Study 0030, 'North American' trial and Study 0027, 'TARGET' trial (Tamoxifen or Arimidex™ Randomized Group Efficacy and Tolerability) performed in Europe, Australia, New Zealand, South America and South Africa) (Nabholtz et al, 2000; Bonneterre et al, 2000). Both studies were similarly designed to allow for subsequent combined analysis to increase the statistical power of the observations made, while individually they were suitably powered to demonstrate equivalent efficacy and tolerability of the different agents. Patients entering the study were either newly diagnosed with advanced breast cancer or had progressed following wing prior treatment of early disease. A drug-free period of at least 12 months was required for those patients receiving prior adjuvant tamoxifen therapy. Hormone receptor status of the patients was determined, and patients were randomized to receive either anastrozole 1 mg/day, or tamoxifen 20 mg/day. The primary end-point that was assessed was time to progression, with objective response, clinical benefit (complete response + partial response + stable disease ≥24 weeks) and tolerability also measured.

Results from the 'North American trial', in which the majority (89%) of patients were known to be oestrogen- or progesterone-receptor-positive, indicated a clear superiority of anastrozole over tamoxifen in terms of time to disease progression (TTP = 11.1 vs 5.6 months for anastrozole and tamoxifen, respectively, $P = 0.005$) and clinical benefit (59% vs 46% for anastrozole and tamoxifen, respectively, $P = 0.0098$). The hazard ratio (1.44, lower one-sided 95% confidence limit = 1.16) also indicated that patients receiving anastrozole displayed a 44% longer disease-free survival period than those in the tamoxifen arm.

In the larger, TARGET study (Bonneterre et al, 2000), only 45% of the study population were known to be oestrogen- or progesterone-receptor-positive. However, anastrozole was at least as effective as tamoxifen in these patients (median TTP 8.2 vs 8.3 months for anastrozole vs tamoxifen, respectively). When data from both studies were combined in a retrospective analysis of those patients known to be hormone-receptor-positive, anastrozole was again shown to be significantly superior to tamoxifen (median TTP 10.7 vs 6.4 months for anastrozole and tamoxifen, respectively, two-sided $P = 0.022$, Figure 3). Furthermore, a smaller study that included individuals who had hormone-sensitive tumours but who had not previously received hormonal therapy, was reported recently. The data showed a significant improvement in TTP (10.6 months in the anastrozole arm vs 5.3 months in the tamoxifen group, $P < 0.05$) similar to that observed in the North

 *© 2001 Cancer Research Campaign*

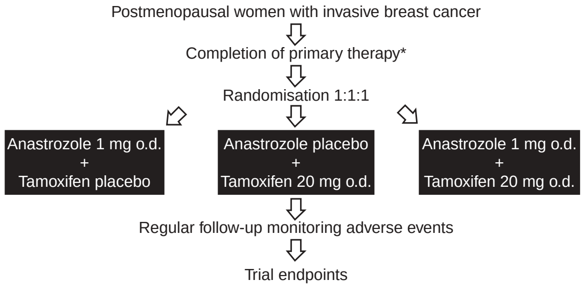

**Figure 4** The ATAC trial study design (*surgery + radiotherapy + chemotherapy. Patients may start trial therapy while still receiving radiotherapy)

American study, and a survival advantage for anastrozole compared with tamoxifen for first-line treatment (Milla-Santos et al, 2000).

In the two pivotal first-line trials, both anastrozole and tamoxifen were shown to be well tolerated with approximately 5% of patients in both treatment arms being withdrawn from the study as a result of adverse events, and 2% of these withdrawals considered to be drug-related. However, when predefined adverse events were considered in the combined analysis of both trials (i.e. those that are predicted to occur due to the known pharmacology of either agent), significantly fewer thromboembolic events (3.6% vs 6.5%, $P = 0.043$, not adjusted for multiple comparisons) (AstraZeneca, data on file) and less vaginal bleeding (1% vs 2.2%) were observed in patients receiving anastrozole (Buzdar et al, 2000). Moreover, data from the combined analysis indicates no clinically significant effect on blood lipid profiles after 108 weeks' follow-up (Dewar et al, 2000), or any significant difference in the frequency of bone fractures in either treatment arm (2.2% in the anastrozole group vs 2.9% in the tamoxifen group) (AstraZeneca, data on file). These data are particularly reassuring given the concern that has been raised regarding the powerful oestrogen-suppressing effects of the third-generation aromatase inhibitors, such as anastrozole. The North American and the TARGET studies indicate that anastrozole is superior to tamoxifen in postmenopausal women with advanced disease who are known to be hormone-receptor-positive. Subsequently, anastrozole has now received approval for first-line treatment of advanced breast cancer in postmenopausal women in many countries.

### ANASTROZOLE IN EARLY BREAST CANCER

The clinical experience of anastrozole in the treatment of advanced disease, outlined above, clearly highlights that this agent also meets the necessary criteria for evaluation as an effective adjuvant therapy in postmenopausal women. These criteria include superior efficacy over existing adjuvant agents, improved tolerability and easy and convenient dosing.

Given anastrozole's superior efficacy compared with tamoxifen in advanced disease, it was postulated that anastrozole would be superior in the treatment of early disease. Tolerability assumes greater importance in the adjuvant setting when the duration of therapy extends to 5 years. Anastrozole's improved side-effect profile compared with tamoxifen particularly in terms of thromboembolic events and vaginal bleeding, make it an attractive candidate for such use.

### The ATAC trial

The ATAC trial was designed to determine whether long-term anastrozole therapy may be an alternative or a complement to tamoxifen in the adjuvant setting. The combination arm of the study may determine whether oestrogen blockade by two different mechanisms – suppression of oestrogen synthesis by aromatase inhibition and prevention of oestrogen binding to its receptor by tamoxifen – provides additional benefits over either agent used alone.

The ATAC trial is the largest single adjuvant breast cancer trial ever to be conducted in postmenopausal women with early breast cancer. Recruitment is now complete with over 9366 patients included, from 380 centres in 21 countries. The trial is a randomized double-blind, three-arm study (Figure 4), statistically powered to demonstrate equivalence of anastrozole and tamoxifen, and superiority of the combination arm over tamoxifen alone, on the primary end-points (recurrence-free survival and tolerability) (Baum, 1999). Patients were randomized to receive appropriate therapy for 5 years following completion of primary surgery. Secondary end-points include time to distant recurrence, time to death and incidence of new breast cancer primaries.

Theoretical concerns that have been raised include the potential detrimental effects on quality of life, bone mineral density and impact on the endometrium. In response, a number of sub-protocols of the ATAC trial were established which compare the long-term effects of all three treatments concerning these specific parameters and will also supply important additional information on quality of life, endometrial histology and pharmacokinetics.

The ATAC trial is the most advanced adjuvant aromatase inhibitor study currently underway and will be the first trial to report on the comparative effects of an aromatase inhibitor and tamoxifen in this setting. The large size of the study will also provide important additional information regarding international and regional variations among the baseline and demographic data. There is little doubt that the power of the ATAC study will be important in answering the key question of whether an aromatase inhibitor is superior to tamoxifen in the adjuvant setting.

### FUTURE PROSPECTS

In the long term, prospects may also exist for using anastrozole as a neo-adjuvant agent. Two clinical trials are already underway to determine whether or not anastrozole provides a benefit in such a setting – the Immediate Preoperative Arimidex, Tamoxifen, or Combined with Tamoxifen (IMPACT) trial and the Preoperative Arimidex Compared with Tamoxifen (PROACT) trial.

The new indication for tamoxifen in reducing breast cancer incidence in high-risk women that has been approved in the USA suggests that anastrozole may have a role in the preventive setting and is likely to be an additional focus of future studies.

### CONCLUSION

Clinical evidence is growing in support of aromatase inhibitors as an important alternative endocrine agent in the management of postmenopausal women with hormone-sensitive advanced breast cancer. Consequently, they are now becoming established as treatments of choice over other established endocrine therapies as both first- and second-line agents for women with advanced breast cancer. Anastrozole appears more selective in the clinical setting

© 2001 Cancer Research Campaign 

with respect to adrenal steroidogenesis and lack of androgenic side-effects. This favourable profile, together with its superiority over tamoxifen in advanced breast cancer, make it a suitable agent for assessment of its effectiveness in the treatment of early disease. The results of the ATAC trial will determine if its superiority over tamoxifen in advanced disease will also translate into the early disease setting in postmenopausal women.

### REFERENCES

Bajetta E, Zilembo N, Noberasco C, Martinetti A, Mariani L, Ferrari L, Buzzoni R, Greco M, Bartoli C, Spagnoli I, Danesini GM, Artale S and Paolini J (1997) The minimal effective exemestane dose for endocrine activity in advanced breast cancer. *Eur J Cancer* **33**: 587–591

Bajetta E, Zilembo N, Dowsett M, Guillevin L, Di Leo A, Celio L, Martinetti A, Marchiano A, Pozzi P, Stani S and Bichisao E (1999) Double-blind, randomised, multicentre endocrine trial comparing two letrozole doses in postmenopausal breast cancer patients. *Eur J Cancer* **35**: 208–213

Baum M (1999) Use of aromatase inhibitors in the adjuvant treatment of breast cancer. *Endocr Relat Cancer* **6**: 231–234

Bonneterre J, Thürlimann B, Robertson JF, Krzakowski M, Mauriac L, Koralewski P, Vergote I, Webster A, Steinberg M and von Euler M (2000) Anastrozole versus tamoxifen as first-line therapy for advanced breast cancer in 668 postmenopausal women: results of the tamoxifen or arimidex randomized group efficacy and tolerability study. *J Clin Oncol* **18**: 3748–3757

Buzdar AU, Jonat W and Howell A (1998) Anastrozole versus megestrol acetate in the treatment of postmenopausal women with advanced breast carcinoma: results of a survival update based on a combined analysis of data from two mature phase III trials. Arimidex Study Group. *Cancer* **83**: 1142–1152

Buzdar A, Nabholtz JM, Robertson JF, Thurlimann B, Bonneterre J, von Euler M, Steinberg M and Webster A (2000) Anastrozole (Arimidex) versus tamoxifen as first-line therapy for advanced breast cancer in postmenopausal women? Combined analysis from two identically designed multicenter trials. *Proc ASCO* **19**: 154A (Abstr 609D)

Dewar J, Nabholtz JM, Bonneterre J, Buzdar A, Robertson JFR, Thurlimann B and Clack G (2000) The effect of anastrozole (Arimidex™) on serum lipids – data from a randomized comparison of anastrozole vs tamoxifen in postmenopausal women with advanced breast cancer. *Breast Cancer Res Treat* **64**: 51 (Abstr 164)

Early Breast Cancer Trialists' Collaborative Group (1992) Systemic treatment of early breast cancer by hormonal, cytotoxic or immune therapy. 133 randomized trials involving 31,000 recurrences and 24,000 deaths among 75,000 women. *Lancet* **339**: 1–15

Early Breast Cancer Trialists' Collaborative Group (1998) Tamoxifen for early breast cancer: an overview of the randomised trials. *Lancet* **351**: 1451–1467

Fisher B, Costantino JP, Wickerham DL, Redmond CK, Kavanah M, Cronin WM, Vogel V, Robidoux A, Dimitrov N, Atkins J, Daly M, Wieand S, Tan-Chiu E, Ford L and Wolmark N (1998) Tamoxifen for prevention of breast cancer: report of the National Surgical Adjuvant Breast and Bowel Project P-1 Study. *J Natl Cancer Inst* **90**: 1371–1388

Gale KE, Andersen JW, Tormey DC, Mansour EG, Davis TE, Horton J, Wolter JM, Smith TJ and Cummings FJ (1994) Hormonal treatment for metastatic breast cancer. An Eastern Cooperative Oncology Group phase III trial comparing aminoglutethimide to tamoxifen. *Cancer* **73**: 354–361

Geisler J, Bernsten H, Ottestad L, Lindtjorn B, Dowsett M and Lonning PE (1999) Neoadjuvant treatment with anastrozole ('Arimidex') causes profound suppression of intra-tumor estrogen levels. *Proc ASCO* **18**: 82a (Abstr 311)

Johnston JO and Metcalf BW (1984) Novel Approaches to Cancer Chemotherapy pp 307–328. London: Academic Press

Jones S, Vogel C, Arkhipov A, Fehrenbacher L, Eisenberg P, Cooper B, Honig S, Polli A, Whaley F, di Salle E, Tiffany J, Consonni A and Miller L (1999) Multicenter, phase II trial of exemestane as third-line hormonal therapy of postmenopausal women with metastatic breast cancer. Aromasin Study Group. *J Clin Oncol* **17**: 3418–3425

Kaufmann M, Bajetta E, Dirix LY, Fein LE, Jones SE, Zilembo N, Dugardyn JL, Nasurdi C, Mennel RG, Cervek J, Fowst C, Polli A, di Salle E, Arkhipov A, Piscitelli G, Miller LL and Massimini G (2000) Exemestane is superior to megestrol acetate after tamoxifen failure in postmenopausal women with advanced breast cancer: results of a phase III randomized double-blind trial. The Exemestane Study Group. *J Clin Oncol* **18**: 1399–1411

Michaud LB and Buzdar AU (1999) Risks and benefits of aromatase inhibitors in postmenopausal breast cancer. *Drug Safety* **21**: 297–309

Milla-Santos A, Milla L, Rallo L and Solano V (2000) Anastrozole vs tamoxifen in hormonodependent advanced breast cancer. A phase II randomised trial. *Breast Cancer Res Treat* **64**: 51 (Abstr 173)

Nabholtz JM, Buzdar A, Pollak M, Harwin W, Burton G, Mangalik A, Steinberg M, Webster A and von Euler M (2000) Anastrozole is superior to tamoxifen as first-line therapy for advanced breast cancer in postmenopausal women: results of a North American multicenter randomized trial. Arimidex Study Group. *J Clin Oncol* **18**: 3758–3767

Plourde PV, Dyroff M, Dowsett M, Demers L, Yates R and Webster A (1995) ARIMIDEX: a new oral, once-a-day aromatase inhibitor. *J Steroid Biochem Mol Biol* **53**: 175–179

Plourde PV, Dyroff M and Dukes M (1994) Arimidex: a potent and selective fourth-generation aromatase inhibitor. *Breast Cancer Res Treat* **30**: 103–111

Smith IE, Harris AL, Morgan M, Gazet JC and McKinna JA (1982) Tamoxifen versus aminoglutethimide versus combined tamoxifen and aminoglutethimide in the treatment of advanced breast carcinoma. *Cancer Res* **42**:(Suppl 8): 3430S–3433S

Yates RA, Dowsett M, Fisher GV, Selen A and Wyld PJ (1996) Arimidex (ZD1033): a selective, potent inhibitor of aromatase in postmenopausal female volunteers. *Br J Cancer* **73**: 543–548

 *© 2001 Cancer Research Campaign*
